# Meetings of Societies

**Published:** 1936

**Authors:** 


### Meetings of Societies

---

### ℞ GALENICALS ℞

GALENICALS had a successful summer term. Membership and interest continue to increase.

On May 7th Professor Lockhart's film on "Anatomical Movements in Living Acrobats" was exhibited.

On May 21st Professor Brocklehurst gave a very interesting lecture on "High Altitude Problems."

Excursions were made by members to the laboratories of Messrs. Glaxo Ltd., Greenford, where the synthesis of vitamins was found to be of particular interest, and to the works of Messrs. Boots Ltd., Nottingham.

The programme has now been completed for the forthcoming autumn term. All meetings will be held in the Physiology Lecture Theatre on Thursday nights at 8.15 p.m.

The first is on October 15th, when Mr. E. W. Hey Groves will speak on "Some Critical Moments in my Career."

The second is on November 5th, when Dr. Macdonald Critchley will speak on "Peter Kürten, the Düsseldorf Mass Murderer."

The third will be on November 26th, at which a debate: "That in the opinion of this house the existing system of medical teaching needs immediate revision," will take place, in which both staff and students will be able to air their views.

The committee wishes to point out that duly-qualified medical practitioners and medical students may attend these meetings as guests, and Galenicals would welcome any members of the Bristol Medico-Chirurgical Society. Application should be made to the Secretary, Mr. L. Willoughby, 1B Elton Road, Tyndall's Park, Bristol 8, who will be pleased to send admission cards.

The committee would also like to point out that associate membership of Galenicals is open to all teachers in, and all qualified ex-*medical* students of, the Bristol Medical Faculty, at a subscription of 5s. for life, and it is hoped that in time many will join.

